# Supplementary material for: Are carbon emissions trading and green financial instruments synergistic? -Comprehensive quantitative research based on content analysis
Source: PLoS One. 2024 Mar 7;19(3):e0298601. doi: 10.1371/journal.pone.0298601 (PMC10919601; doi:10.1371/journal.pone.0298601)
Supplement: S1 Data — (ZIP) [file pone.0298601.s001.zip › Data/地级市Z_F工作报告.zip!/║ú─╧/║ú┐┌╩╨/└┤╫╘╣1⁄2╓┌║┼í╛┬φ┐╦╩2╛▌═°í┐_2015.docx]

各位代表：

　　现在，我代表市人民政府向大会报告工作，请予审议，并请各位政协委员和其他列席人员提出意见。

　　一、2014年工作回顾

　　过去一年，面对错综复杂的经济形势和两次强台风自然灾害，在省委、省政府和市委的坚强领导下，在市人大、市政协的有力监督和大力支持下，我们紧紧依靠全市人民，认真学习贯彻党的十八大、十八届三中、四中全会和习近平总书记一系列重要讲话精神，坚持“稳中求进、改革创新”的主基调，主动适应经济发展新常态，着力稳增长、促改革、调结构、惠民生、强管理，攻坚克难，务实创新，经济社会发展持续向好，较好地完成了市十五届人民代表大会第五次会议确定的目标任务。全市生产总值1005.5亿元，增长9.2%;来自海口的公共财政收入267.7亿元，增长11.2%，地方公共财政预算收入100.1亿元，增长15.4%;固定资产投资821.5亿元，增长26.5%;社会消费品零售总额541.3亿元，增长10.5%;城镇常住居民人均可支配收入26,530元，增长9.6%;农村常住居民人均可支配收入10,630元，增长12.4%;居民消费价格涨幅2.2%;城镇登记失业率1.37%。全市生产总值突破千亿元大关，地方公共财政预算收入跨上百亿元台阶，农村常住居民人均可支配收入过万元，实现“千百万”突破。

　　(一)主动适应新常态，经济平稳健康发展

　　积极应对经济下行压力，把扩投资、促消费、调结构作为主抓手，着力解决经济运行的困难和问题。

　　重点项目推进力度加大。紧紧抓住项目建设“牛鼻子”，把推进重点项目建设作为拉动经济增长的引擎。扎实开展“项目建设年”活动，落实“六个一”责任制，强化“一竿子插到底”工作机制，深入一线，走访重点项目，集中解决问题。全年省市重点项目实现投资446亿元，其中，年度投资过10亿元的项目11个、过亿元的项目48个，有望连续第三年获得省政府重点项目上台阶奖。

　　消费需求持续扩大。开展农副产品、旅游和房地产促销活动50多场次，建立琼北旅游交易大平台，实现旅游直接总收入142亿元，增长18.2%;“冬交会”海口签约订单26.3亿元，增长5.2%;全年销售商品房337.1万平方米。扩建美兰机场离岛免税店，免税商品全年销售9.2亿元，增长52.5%，离岛免税政策效应进一步放大。启动电子商务促进工程，建成江东电子商务创业园，培育本土电子商务平台9家。深入推进“万村千乡市场工程”和“新网工程”建设，升级改造农贸市场4家。落实促进信息消费、健康服务等政策，启动医养结合试点，形成新的消费热点。全市实现零售业销售额604.5亿元。

　　产业发展质量提升。针对经济下行压力和产业结构转型优化的需求，及时出台促进企业发展十项举措、灾后重建二十条、灾后稳增长三十条，壮大实体经济，加快产业发展。

　　服务业发展加快。第三产业增加值增长11.3%。

　　旅游、文体和会展融合发展。东寨港红树林旅游区、冯小刚电影公社正式营运，骑楼历史文化街区业态调整初见成效，旅游新业态效益显现;全年举办各类文化赛事20多项，高尔夫世界明星赛、海南岛欢乐节、换花节等品牌活动影响力不断扩大;举办2014中国城市规划年会等规模会展活动208场次，国际会展中心开馆率达到45%，高于全国平均水平。全年接待国内外过夜游客1131万人次，被评为全国旅游标准化示范城市。交通运输业增长25.3%，成为推动经济增长的重要动力。驻市金融机构累计达101家，金融业增长17%，占GDP比重进一步提升。启动远大等大型商业综合体建设，加快推进中国华信等项目建设，商贸服务业层级进一步提升。

　　工业产业结构逐步优化。在电力短缺、市场疲软、产值下降的严峻形势下，工业增加值增长3.7%，产业效能进一步提升。医药、机电、食品和农副产品加工等产业持续增长，光伏产业基础不断夯实，新材料产业业态初现。美安生态科技新城一期完成投资28亿元，云龙产业园基础设施及配套二期建设有序推进，园区基础设施和配套服务进一步完善;落实工业企业扶持资金3.2亿元，协调501家中小微企业获得贷款24.95亿元。

　　农业发展企稳回升。大灾之年完成农业增加值54.6亿元。全年建设蔬菜大棚600亩、改造冬季瓜菜基地1.1万亩，新增常年蔬菜基地9000亩、本地叶菜种植1500亩，全市瓜菜种植面积达到42万亩，总产量56万吨。新建标准化畜牧养殖小区34个、生态循环农业示范基地15个，罗牛山10万头养猪场异地技改开工建设，柏盈兰花产业园二期加快推进。新建区、镇、村三级农产品质量安全检测机构144个。

　　(二)深化改革开放，激发内生动力

　　各领域改革稳步推进。精减行政审批服务事项713项，清理行政审批中介机构185家，推出审批便民服务新举措107项，市、区、镇(街)三级所有事项实现网上申报。推进工商登记改革，新登记企业1.47万家，增长50.7%。实行优质初中学校划片就近入学和指标到校招生改革。启动市属公立医院取消药品加成改革，“先看病后付费”诊疗服务模式试点扩大到5家公立医院。深化财税体制改革，推进“营改增”扩围，全市企业税赋减负近6亿元;建立完整预算管理体系，部门预决算网上公开。清理农村土地对外发包“三过”问题441宗，完成4个试点镇农村土地承包经营权确权登记。推动海口港与洋浦港小铲滩的港航资源重组，为打造海口在环北部湾区域航运枢纽地位奠定了基础。顺利承接三江农场移交属地管理，完成市属事业单位预分类和食品药品监督管理体制改革。

　　扩大开放取得新成效。抢抓“21世纪海上丝绸之路”战略机遇，新开辟国际及地区空中航线11条，新增国际友城3个。加强琼桂粤区域港口通关合作，签署“三地七港”环北部湾合作备忘录。综合保税区国际商品展示交易中心建成开业，海口港(秀英港区)被列为国家汽车整车进口口岸。加大招商引资力度，全年共举办和参加招商推介活动16场，签订项目23个，落地率56.5%，引进保利、林安、万达等13家企业，全年招商引资到位资金449亿元。外贸进出口总额34亿美元。泛珠“9+2”区域合作、省会经济圈发展协作机制日益深化，成效显现。成功举办2014年世界城市和地方政府联合组织(UCLG)世界理事会、亚洲市长论坛(AMF)第四次全体会议和海口首届国际友城市长会议，海口国际化水平进一步提升。

　　(三)聚焦城市管理，提升城市品位

　　城市管理力度加大。持续深入开展“城市管理年”活动，整合多部门资源，成立市级公共安全联动指挥中心，城市一体化综合管理体系初步建立。提升数字化信息系统，建立市区镇(街)信息共享、跨部门联动的“数字城管”信息平台。强化城市应急管理，出台《防御强台风应急总预案》。制定违法建筑分类处置和责任追究办法，开展拆后土地利用试点，全年拆除违建142万平方米。强化交通专项整治，加大投入，优化道路交通组织，大力整治“黑车”非法营运，平稳退市超标电动车31万辆，公交都市创建稳步推进，市民出行环境有所改善。狠抓汽车尾气排放、烧烤摊点、工地扬尘整治，淘汰黄标车、老旧车1.18万辆，空气质量稳居全国74个重点城市第一。深入开展“绿化宝岛”大行动，植树造林2.01万亩;编制《海口市环境总体规划》，对东寨港红树林湿地实施立法保护，规划管理范围扩大到12万亩。坚持集约节约利用土地，处置闲置土地120宗、6394亩。推动多规衔接，开展城市总体规划实施评估。中心城区“四沟四湖”和美舍河污水截流并网工程开工建设，滨江路3个排涝泵站建成使用，改造滨海西路、海秀东路、和平南路等积水点8个，建成街心小游园5个，完成金鼎路、长兴路等道路贯通工程7条，提前一年实现打通32条“断头路”的目标。

　　社会治理不断加强。深化社区网格化管理体制改革，城区实现网格全覆盖。美兰区被评为“全国和谐社区建设示范城区”。加强农资、食品药品等重点商品市场监管，严厉打击生产经营违法违规行为，市场环境进一步净化。大力开展“大排查、大调解、大治理”专项活动，化解矛盾纠纷7128宗，办理法律援助案件8537件。大力推行领导干部接访下访、信访积案包案化解制度，协调解决了房产办证、土地纠纷、拆迁安置等一批群众反映强烈的历史遗留问题。严格落实“党政同责、一岗双责”，安全生产形势平稳。依法严厉打击各类违法犯罪活动，社会治安保持稳定。

　　(四)统筹城乡发展，推进新型城镇化进程

　　棚户区改造提速。发挥棚改对投资、消费的双拉动作用，不断完善“政府让利、企业微利、百姓得利”的棚改模式，上贤、滨涯、五源河等棚改项目进展顺利，启动1.3万户棚户区(城中村)改造，惠及4.2万人，占全省城中村改造任务的64.3%。开工建设12,984套安置房，棚改片区人居环境和生活质量得到进一步改善。

　　示范镇建设扎实推进。百个统筹城乡示范项目进展顺利。云龙镇镇墟街景立面改造竣工，镇墟改造配套工程基本完成，建成乡镇规划展览馆和乡镇燃气管道，“六权”确权颁证工作全面推进，被评为“2014海南十大最美小镇”。演丰镇老豆藤商业街、瑶城新型农村社区基本建成，引入供气站、红树林商业街、海口(演丰)现代农业产业园一期等6个社会投资项目。

　　农村基础设施进一步完善。改建县道砂土路68公里，新建农村公路56公里。25座小Ⅱ型病险水库除险加固竣工使用，南渡江3个防洪工程即将完工。改造农村危房3500户。完成农村安全饮水项目31个，解决了4.1万人安全饮水问题。深入整治农村环境卫生，“户分类、村收集、镇运转、市处理”的农村垃圾收运体系覆盖1279个自然村，覆盖率达73%。秀英区石山镇美社村获得“2014海南十大最美乡村”殊荣。

　　(五)全力保障民生，群众生活不断改善

　　全年民生支出102.5亿元，同比增长15.3%，占地方公共财政支出的70%。受强台风影响，金牛湖水环境净化美化工程、沙坡—白水塘公园一期起步区春节前完工，省市其它为民办实事事项全部完成。

　　千方百计扩大就业，新增城镇就业5.6万人，农村富余劳动力转移就业1.2万人。加大社会保障力度，企业退休人员月人均养老金提高到1791元，新农保基础养老金标准提高到120元。实施被征地农民社会养老保险，城乡居民养老保险参保缴费率达97.9%。开展“救急难”工作试点，发放城乡低保救助资金1.19亿元;修建农村幸福院33个，3家乡镇敬老院投入使用。进一步扩大保障性住房保障面，配售配租4046套，发放租赁补贴1365万元。投入1.6亿元开展扶贫攻坚，有102个贫困自然村脱掉“贫困帽”。建立“菜篮子”工程建设联席会议制度，多措并举解决“菜篮子”问题，全力保障物价稳定。

　　加大教育投入，建成30所乡镇公办幼儿园;加快义务学校建设，美苑小学、玉沙学校建成;整合麻雀学校，美兰实验学校投入使用;美兰区通过义务教育基本均衡发展国家评估认定，琼山区义务教育基本均衡发展建设基本完成;龙华区在全省率先实行学区化管理模式改革。提高医疗服务水平，实施基本和重大公共卫生服务项目，开展城乡医院对口帮扶、社区卫生契约试点，实施病种限价、总额预付等新农合支付方式改革，123.4万人次享受新农合补偿2.2亿元。启动“单独二孩”政策，综合治理出生人口性别比，人口自然增长率控制在10%。以下。

　　国防动员、人民防空、优抚安置、防震减灾扎实推进，获得全国防震减灾工作综合考核先进市、省双拥模范城等荣誉称号。统计、口岸、粮食、气象、保密、档案、史志、民族宗教、外事侨务等工作取得新成绩，工会、青少年、妇女儿童、老龄、残疾人、慈善等事业取得新进步。

　　(六)携手抗风救灾，灾后重建取得重大胜利

　　过去的一年，我市遭受历史罕见的两次强台风袭击。面对严重自然灾害，在省委、省政府和市委的坚强领导下，我们始终把确保人民群众生命财产安全放在首位，紧急动员、迅速行动，转移、安置群众25.6万人，全力恢复生活生产秩序。4750户受损房屋全部修缮，323户倒损房屋重建全部完成，春节前可搬迁入住新居。损毁基础设施修复进展顺利，农业、渔业和旅游业等已恢复正常生产。在这场抗风救灾中，全市人民、部队官兵、各条战线广大干部职工众志成城，不畏艰险，连续作战，涌现出了“中国好人”环卫工人邓育军等一批先进典型，为抗风救灾、重建家园提供了源源不断的精神力量。

　　(七)转变工作作风，加强政府自身建设

　　自觉接受人大法律监督和政协民主监督，积极听取各民主党派、工商联、无党派人士及社会各界的意见和建议。办理市人大代表建议344件、市政协委员提案331件，办结率100%。坚持依法行政，加强执法监督，强化行政复议，依法行政考核连续六年全省第一。全面开展党的群众路线教育实践活动，集中清理公务用车和办公用房，“三公”经费支出下降23.3%，解决了一批群众反映强烈的突出问题。开办电视问政《亮见》栏目，全面实施党风政风行风公开述职评议。加强廉政建设，开展“不干事、不担事”和涉农资金、土地出让金审计等突出问题专项整治，立案查处“两不”行为案件4宗，问责20人，党纪政纪处分3人。

　　各位代表!过去一年的成绩来之不易，这是省委、省政府和市委坚强领导的结果，是全市人民团结拼搏的结果。在此，我代表市人民政府，向全市人民致以崇高的敬意!向人大代表、政协委员，向各民主党派、工商联、各人民团体和社会各界人士，向驻市部队、武警官兵和公安司法干警，表示衷心的感谢!

　　同时，我们也清醒地看到发展中还存在不少困难和问题：经济下行压力依然存在，产业结构不合理的问题还未得到根本解决，财政增收压力较大;城市防灾减灾和城市管理还需加强，公共安全应急处置能力有待进一步提升;部分政府工作人员服务意识、办事效率和执行能力还不适应发展需要，不作为、不担责、为政不廉等现象仍然存在。对此，我们必须高度重视，认真对待，有效解决。

　　二、2015年工作安排

　　今年是全面深化改革的关键之年，是全面推进依法治国的开局之年，也是“十二五”规划的收官之年、“十三五”规划的谋篇之年。做好今年工作，具有十分重要的意义。

　　新常态下海口的发展面临难得机遇和有利条件：一是海口作为国家“21世纪海上丝绸之路”战略支点城市，面临重大战略机遇。二是省委、省政府全方位支持“首善之城”，加快以海口为中心的省会经济圈建设，为海口加快发展进一步指明了方向、增添了动力。三是这些年我们做了许多打基础、管长远的工作，积蓄了后劲，为今后的发展奠定了坚实基础。

　　今年政府工作的总体要求是：全面贯彻落实党的十八大、十八届三中、四中全会，习近平总书记系列重要讲话精神和省委六届七次全会精神，按照市委十二届八次全会的部署，坚持稳中求进、依法治市，以提高经济发展质量和效益为中心，主动适应经济发展新常态，着力深化改革扩大开放，着力转方式调结构，着力培育新的经济增长点，强化社会治理，推进民生改善，确保经济平稳健康发展和社会和谐稳定。

　　今年经济社会发展的主要预期目标是：全市生产总值增长8.5%左右;地方公共财政收入增长10%左右;固定资产投资增长16%左右;社会消费品零售总额增长12%左右;城镇常住居民人均可支配收入增长9.5%左右，农村常住居民人均可支配收入增长10%左右;城镇登记失业率控制在3%以内;居民消费价格涨幅控制在4%以内;全面完成省下达的“十二五”节能减排控制目标。

　　实现上述目标，必须扎实做好以下工作：

　　(一)加快推进改革开放，争创体制机制新优势

　　积极推进各项改革。深化行政体制改革，继续减少行政审批、非行政许可和行政审批前置事项，推动部门审批办全部进驻市政务中心，推出第三批便民措施，确保审批事项网上申报和办理率达80%以上，全程网上审批率达40%以上。承接好省级下放行政审批事项，深化强区扩权。启动演丰镇行政管理体制改革国家试点。推动公共资源交易平台整合。推进不动产登记制度改革。推进国资国企改革，完善分类监管体系和考核体系，推动国有企业改革3个试点，筹建国有资本投资公司、国有资本运营公司，适时启动1家国有企业混合所有制改革试点和国企领导人薪酬制度改革。深化财税体制改革，编制预算补充项目和中长期财政规划，建立跨年度预算平衡机制。进一步完善政府购买公共服务机制，建立健全专项转移支付定期评估和退出机制、财政支出绩效评价体系，提高财政资金使用效率。规范政府债务管理，将政府性债务收支在内的政府全部收支纳入预算，建立债务风险预警机制，分门别类消化债务存量。深化投融资体制改革，制定民间投资开放领域和项目清单，出台非公有制企业进入特许经营领域具体办法。启动PPP项目试点，探索财政对企业短期股权投资。采取“政保贷”、“拨改投”、创投基金等方式，发起设立1-2支产业扶持基金，优化财税支持经济发展体系。推进城乡户籍制度、社会保障制度等改革。

　　积极扩大对外开放。主动融入国家发展战略，努力打造“21世纪海上丝绸之路”重要支点城市。加快与北部湾、泛珠三角等区域合作，发挥环北部湾港口合作协调机制作用，推动琼桂粤“三地七港”大通关合作。深化省会经济圈合作，通过产业对接、合作、转移，构筑跨行政区域的产业链。以综保区为基础，马村港、临空产业园空港、美安生态科技新城为组团，探索和实践自贸区可复制改革试点经验。推动综保区与上海钻石交易所通关一体化试点，加快汽车整车进口口岸建设，争取综保区国际大宗商品交易中心获批建设。发布外商投资负面清单，探索融资租赁、跨境电商等新型业务。努力扩大进出口规模，保持外贸稳定增长。

　　(二)培育新的经济增长点，保持经济平稳增长

　　推动服务业提档升级。推进旅游、文体、会展融合发展，延伸旅游发展内涵，培育养生度假、影视动漫、邮轮游艇、低空飞行等旅游新业态;深化琼北旅游合作，加快旅游吸引物建设，申报创建1家5A级景区，争取新增1家4A级景区，启动“琼北到城”项目建设;进一步挖掘地方特色文化旅游资源，定期定址办好观澜湖世界杯女子高尔夫锦标赛、海口国际沙滩马拉松赛等品牌文化赛事活动;启动“海上丝路体验游”。大力发展规模品牌商务会展，全年举办会展活动200场次以上。争取旅游直接总收入有新突破。优化房地产投资结构，按照“消化存量、优化增量”的思路，进一步控制岸线房地产和普通商品住房开发，力争今年旅游地产、产权式酒店、医养结合等经营性地产占比达26%以上。促进商贸物流调整优化，完成主城区商业网点规划修编和计划单列镇商业网点规划编制，启动海口万达广场建设，加快华联、现代美居、海航日月广场、西海岸远大商业综合体建设;结合临空、大印、美安、林安等物流园区建设，抓好城区专业批发市场布局调整。促进金融业稳健发展，做好中小企业集合债发放，推动本土企业上市、上市企业再融资，鼓励和引导金融机构创新涉农信贷产品，加强与泛珠省会城市金融交流合作，推动金融资源优化配置、优势互补。加快发展文化创意产业，推进海口国际创意港建设，着力开发工业设计、广告制作、软件开发、动漫游戏等创意产品。培育发展信息产业，聚焦以移动互联网、物联网、电子商务等为代表的互联网应用产品，落实促进电子商务发展鼓励政策，加快4G网络覆盖普及，建设智慧城市，促进信息消费。加快生活性服务业发展，健全社区生活消费服务网点，促进健康养老、家政服务等产业发展。大力发展总部经济、楼宇经济，吸引世界500强企业、跨国公司、国内知名企业、上市公司进驻海口。

　　狠抓项目建设。制定“重点项目清单”、“工作责任清单”，强化项目协调服务，扎实推进68个省重点项目建设，完成352亿元年度投资计划。开工建设威特电气、先声药业、英利太阳能电池三期等工业项目，秀英港旅游综合体等商业项目，江东大道二期、琼山大道延长线、颜春岭垃圾焚烧发电厂扩建等基础设施项目;加快推进观澜湖旅游区、恒大文化旅游城、东寨港红树林乡村旅游区、长影环球100等文化旅游项目建设。强化招商引资，健全责任目标体系，重点策划引进一批以重大基础设施、棚户区改造为核心的城市建设项目，以新型工业、现代农业、商贸物流、文化旅游等为重点的产业项目，增强发展后劲。

　　壮大新型工业。大力发展医药、汽车制造运输、机电、食品加工、新能源等优势主导产业，延伸产业链条，争取培育1-2个上下游配套、多企业共生的产业集群。加强工业园区建设，全面加快美安生态科技新城一期的融资开发和建设，加大项目落地和推进力度，确保6个以上产业项目入园。鼓励骨干企业开展强强联合、资产重组、上市融资，新增2家以上产值过亿元企业。实施中小工业企业选苗培育成长工程，在担保贷款、贷款贴息、项目申报等方面加大扶持力度，力争4-5家升级为规模以上工业企业，全年规模以上企业工业增加值增长7.5%左右。

　　加快农业发展。继续抓好马坡洋、新坡洋、那力洋二期等常年蔬菜基地建设，稳定冬季瓜菜种植面积，改造1.48万亩标准化菜田，抓好罗牛山10万头生猪异地技改，创建15家标准化畜禽生态养殖小区，增强综合生产能力。制定品牌农业专项规划，申报2个国家地理标志农产品、8个省著名商标和名牌产品。加大对农业龙头企业、农民专业合作社、家庭农场和特色农业的扶持力度，引导工商资本到农村发展现代种养业。积极发展涉农“电商”，用信息化技术缩短农民与市场的距离。加强农村集体“三资”管理。力争完成农村土地承包经营权确权登记颁证，引导和规范土地经营权有序流转，下大力气解决好土地撂荒问题。

　　发展海洋经济。争取《海口市海洋功能区划》获批实施，加快调整海口港功能布局，完善海口港基础设施。推进东海岸如意岛、南海明珠等填岛工程建设。协调推动水域开放，加快发展以邮轮游艇为主的海洋旅游产业。积极培育海洋生物医药和海洋旅游装备制造业。配合做好南海资源开发服务保障工作。

　　实施创新驱动。积极争取国家新兴产业投资引导基金、省创业投资引导基金，加大对各类创新活动的支持力度，推动大众创业、万众创新。大力培育新型交叉产业和创新业态，主动适应生产小型化、智能化、专业化新特征，探索推进农业、旅游、文化、健康、信息等领域的跨界融合发展。加快建立以企业为主体、以市场为导向、产学研相结合的技术创新体系，力争新增高新技术企业5家、市级以上企业技术研发中心2家。

　　(三)加快新型城镇化建设，推动城乡一体发展

　　加快“生态立市”。强化规划引领，编制《生态保护建设规划》，科学构建生产、生活和生态空间格局。落实“谁开发谁保护、谁受益谁补偿”的生态保护长效机制，健全完善投资项目环评、能评制度，加强环保执法，严惩破坏生态环境行为。抓好南渡江流域生态保护，强化两岸管控。依法保护森林资源，抓好海防林等损毁森林资源生态修复，打造一批沿江、沿湖、沿河生态绿化带，全面完成“绿化宝岛大行动”各项任务。加大饮用水水源保护，推进城镇污水管网、垃圾收集及无害化处理设施建设，促进海岸线、河道、湖泊、水库等水资源的综合保护、合理开发和有效利用，提高水域环境质量。强化大气污染治理，加速淘汰黄标车，重点整治机动车排气污染、垃圾焚烧、露天烧烤、建筑施工和裸露地面扬尘，确保空气质量继续保持一流。

　　加快主城区建设升级。继续加快城区道路建设，启动长滨路中段等21个新建道路项目。合力推进西环高铁、定海大桥建设，拓展对外大通道。利用国家地下综合管廊试点城市机遇，完成城市地下管线信息普查，启动老旧管网改造。加快旧城区雨污分流管网改造工作，推进美舍河、大同沟、电力沟、东西湖周边污水管网体系建设。完善城市地下空间开发利用，开展地震小区划规划工作。加快颜春岭生活垃圾渗滤液处理厂和焚烧发电厂二期建设，启动江东生活垃圾综合处理基地建设。力争7月完成世纪公园、万绿园、滨海大道海岸线灾后修复工程。继续推进江东、西海岸、大英山等城市新片区开发。加快公交场站建设，整合优化停车场资源。

　　全面加快棚户区改造。扎实做好棚改项目招商、规划供地、资金统筹、安置拆迁等工作，创新社会资本参与棚改模式，将部分保障性住房房源纳入安置房统筹，提高货币补偿安置占比，激发市场刚性需求。继续推进灵山、五源河、流水坡等片区棚改，完成上贤村、滨涯村安置房回迁使用，新启动8950户棚户区(城中村)改造任务。探索将“三无”小区纳入棚户区改造。

　　推进示范镇建设。坚持规划引领，启动新型城镇化规划战略研究，分档制定镇域基础设施和公共服务设施建设标准。健全区镇两级规划管理机构，聘用一批乡镇规划师，抓好镇墟和居民集中居住点规划，强化农村建房申报管理。抓好统筹城乡试点示范建设，继续加快演丰镇墟改造和云龙示范镇建设，适时启动市级第二批统筹城乡示范镇建设。积极推进“村改居”。加快农村基础设施建设，抓好30万亩土地综合整治、南渡江引水工程建设，建成防洪楼27栋，完成29条农村公路建设，启动79个农村安全饮水项目，基本解决农村饮水安全问题。

　　(四)强化城市管理，提高社会治理能力

　　深化城市综合整治。按照“净化、亮化、绿化、美化”的要求，深入开展环境卫生、建筑垃圾、市容秩序、市政设施、园林绿化、广告招牌和城市信息化管理等“六治一管”专项整治，实现“一年有突破、两年见成效、三年大变样”的目标。重点落实“门前三包”，治理占道经营、乱张贴等行为;启动建筑垃圾资源化再利用和餐厨垃圾无害化处理项目，建设4个建筑垃圾消纳场、8座小型生活垃圾中转站;实施道路绿化提升工程;启动城管综合执法改革试点，加强城管队伍建设，提升城市管理法治化水平。强化交通秩序综合整治，加大车辆超载、违禁行驶、乱停乱放、横穿马路等交通违法违规行为查处，严厉打击“黑车”营运。加大智能交通科技投入，在重要路段设置交通电子导视板。实施清洁家园行动，全面完成农村生活垃圾收运工程二期建设，完善清洁保洁体系。着力整治农贸市场、大型商场及周边环境秩序。

　　加强和创新社会治理。夯实基层基础，改善街道(社区)办公条件，健全基层信息服务平台，巩固和扩大社区管理体制改革成果。完善社会管理体制机制，加强对非公有制组织和社会组织的规范管理。发挥社会团体参与社会治理的作用，完善社区矫正工作机制，强化流动人口、特殊人群管控服务。推进全民学法守法，规范执法行为，强化法律援助，建设“法治海口”。深化商务信用建设，营造诚信环境，规范市场交易主体行为，促进市场公平竞争。强化旅游市场属地管理和联合治理，提升旅游服务水平。加快构建粮食安全保障体系。落实主体责任，高度重视公共安全、生产安全、食品药品安全，突出抓好交通安全、公众聚集场所、中小学校等重点领域的安全监管，确保不发生重特大事故。强化公共安全联动中心综合信息平台运用，进一步健全城市应急管理体系，提高应急处置能力。深化“平安海口”建设，打造立体化社会治安防控体系，依法防范和惩治违法犯罪活动。完善基层信访工作网络，推行网上受理信访制度，建立畅通有序的诉求表达、矛盾调处、权益保障机制。完善重大决策社会稳定风险评估机制和社会预警体系，解决好群众合理诉求。大力弘扬社会主义核心价值观，群众性精神文明创建，加强社会公德、职业道德、家庭美德、个人品德教育，提高全社会文明素质，积极创建“全国文明城市”。坚持军民融合发展，支持军队和国防建设，争创全国双拥模范城“八连冠”。支持工会、共青团、妇联、工商联、侨联、科协、社科联等人民团体发挥桥梁纽带作用。继续做好人防、气象、地震、地质、海事、口岸、统计、档案、史志、保密、外事、侨务、对台、民族宗教等各项工作。

　　(五)加快社会事业发展，持续推进民生改善

　　扩大就业增加收入。统筹抓好企业用工和城乡劳动者就业工作，通过见习补贴鼓励大中专毕业生到小微企业就业，促进棚改居民、失地农民等群体多渠道就业;支持青年、妇女和下岗失业人员自主创业，引导外出务工人员返乡创业，确保城镇新增就业5.5万人以上，确保“零就业”家庭至少有一人就业。健全企业职工工资决定和正常增长机制，提高社会最低工资标准。落实机关事业单位工资制度改革政策。继续开展农民增收行动，整村推进“造血式”扶贫开发，确保城乡居民收入与经济增长同步。

　　健全社会保障体系。实施机关事业单位工作人员养老保险制度改革，继续提高企业退休人员基本养老金和城乡居民基本养老保险基础养老金，抓好被征地农民参加养老保险，提高城乡居民基本医疗保险财政补助标准。提高城乡低保对象补助水平，开展低保对象清理，坚决杜绝“关系保”、“人情保”。推进社会救助主体多元化，引导社会资本兴办社会福利和社区养老机构。完善保障性住房配售配租政策，改造农村危房3500户，满足不同层次低收入群体的住房需求。发展老龄事业，保障妇女权益，加强未成年人保护，关心青少年发展，做好残疾人基本公共服务，支持慈善事业健康发展。

　　坚持教育优先发展。继续实施农村薄弱学校改造工程，确保琼山区通过义务教育基本均衡发展省级评估和国家认定，加快秀英区义务教育基本均衡发展建设，继续优化调整城乡学校布局，启动“10+2”重点项目建设，增加学位供给。积极推进教育信息化。大力发展职业教育，鼓励和支持社会力量办学。

　　强化卫生医疗服务。稳妥推进公立医院改革，积极推进市属公立医院取消药品加成，扩大和完善“先看病后付费”诊疗服务模式，推行床日付费和限费医疗试点。推进城乡医院一体化管理(医疗联合体)服务模式改革，加快城乡医疗卫生服务体系建设。加强卫生医疗人才建设，培养一批基层医疗机构全科医生，提高农村医疗服务水平。开展计生优质服务，综合治理出生人口性别比，提高出生人口素质。

　　狠抓“菜篮子”建设。下大力气保供稳价，建设“菜篮子”蔬菜种植基地3000亩，在蔬菜批发市场、农贸市场和大型超市新设“菜篮子”蔬菜批发专区、销售专用摊位，新增50个便民流动菜(车)网点、社区便民菜摊和连锁便利店“菜篮子”蔬菜专区，新增10个以上平价菜店，设立10个直供直销直营门店，扶持不少于4家生鲜电子商务企业销售“菜篮子”蔬菜产品，建设不少于2家“菜篮子”农产品分拨配送项目，启动2个大中型农副产品综合批发市场建设的前期工作，升级改造11家、新建2家农贸市场，抓好肉、禽、蛋、鱼等主要农产品的生产供应保障，不断健全完善“菜篮子”监测、储备、投放和调运“四位一体”市场供应保障体系。

　　完善公共文化服务。继续实施文化惠民工程，加快五源河文体中心、秀英区文化馆、广播电视村村通等重大公共文化工程建设，新建35个行政村文化活动室、8家农家书屋示范点，完成惠民演出30场、农村公益电影放映3000场。加强文物普查和文化遗产保护，争取五公祠文物保护、海瑞墓扩建改造等工程开工建设。深入开展全民健身活动，办好重大文体赛事，满足人民群众文化消费需求。深入开展扫黄打非，净化社会文化环境。

　　办好十类24件民生实事。一是扩大城市交通覆盖面，改善交通枢纽站间的无缝对接体系，开通和优化12条公交线路;二是改善道路交通环境，改造6处积水点，升级3条道路;三是新建及改建8个市民街心小游园;四是制定城市水体保护蓝线规划，实施水体环境保护治理;五是开展困难群众身份确认及办理户口登记;六是解决15万平方米房产证办理的遗留问题;七是实施农村危房改造、生活垃圾收运设施、农村公路、防洪楼、广播电视村村通建设等工程;八是完善市属公立医院供电安全保障设施、乡镇卫生院基础设施建设;九是新增教育学位5050个，实施校园周边安全监控项目，建成港湾实验小学;十是建成27个社区活动中心、11间农村老年人日间照料中心，实施社区居家养老购买服务。

　　(六)抢抓发展新机遇，科学谋划“十三五”

　　立足“21世纪海上丝绸之路”战略、泛珠三角区域合作和环北部湾城市群建设，在更高层面、更广区域科学谋划未来五年经济社会发展的主要方向和功能定位。坚持多规衔接，注重城市空间布局和空间结构规划，将经济社会发展与优化空间布局融为一体，科学划分城镇、农业、生态三类发展空间。加强项目策划包装，精心谋划储备一批支撑海口未来五年乃至十年以上可持续发展的重大项目，并做好与国家、省“十三五”规划的衔接。

　　三、努力建设法治高效廉洁政府

　　认真贯彻落实党的十八届四中全会、省委六届七次全会和市委十二届八次全会精神，围绕转变政府职能、提高行政效能，加快建设法治高效廉洁政府。

　　加快转变政府职能。制定政府部门权力清单、责任清单和市场准入负面清单，推动政府机构、职能、权限、程序和责任法定化，法定职责必须为，法无授权不可为。厘清政府与市场的边界，该管的坚决管好，不该管的坚决放活，用政府权力的“减法”换取市场活力的“加法”，切实把政府职能转变到公共服务、市场监管和社会治理上来。

　　提高依法行政水平。严格按照法定权限和程序行使权力、履行职责，自觉接受人大法律和工作监督，主动接受政协民主监督，优质高效办理人大代表建议和政协提案，认真听取和采纳各民主党派、工商联、人民团体和社会各界意见，接受新闻媒体和社会公众监督。坚持依法科学民主决策，完善重大决策合法性审查和风险评估机制。加强行政程序立法，提高政府立法质量和水平。规范行政裁量权，加强执法监督检查，明确各类行政执法的职责、流程和标准，强化部门监管执法信息共享，在城市管理、食品药品、工商质检、安全生产等领域探索综合执法，用法律提高市场监管的权威性、有效性。更加注重政务公开，规范政府工作流程，加强电子政务建设，让权力接受监督、在阳光下运行。

　　加强行政效能建设。巩固和扩大党的群众路线教育实践活动成果，持之以恒反“四风”，将作风建设常态化、制度化。强化行政效能监察，深入开展“庸懒散奢贪”、“吃拿卡要拖”、“不干事、不担事”等专项整治，深化政风行风民主评议，持续加大执纪问责力度，倒查追责，着重解决干部作风不实、主动服务意识不强等问题，以实干清正的作风推进各项工作的落实。

　　打造廉洁自律政府。坚持用制度管权管事管人，强化廉洁从政意识。坚持勤俭办一切事业，降低行政运行成本，用政府的紧日子换取群众的好日子。认真履行“一岗双责”，强化党风廉政建设主体责任，切实加强对行政审批、招投标、土地出让、工程建设、政府采购、国有资产产权交易等重点领域和关键环节的监管，突出抓好财政预决算、公共资源配置和重大项目建设的审计监督，努力从源头上预防腐败。

　　各位代表!人民赋予重托，实干推动发展。全面完成今年各项经济社会发展任务，需要全市上下同心协力、埋头苦干。让我们更加紧密团结在以习近平同志为总书记的党中央周围，在省委、省政府和市委的坚强领导下，紧紧依靠全市人民，凝心聚力，真抓实干，以一天也不耽误的精神，为科学发展、绿色崛起，建设“四宜三养”最精最美省会城市和海南“首善之城”而努力奋斗!
